# A sagittally confined high-resolution spectrometer in the ‘water window’

**DOI:** 10.1107/S160057751800468X

**Published:** 2018-04-25

**Authors:** Zhuo Li, Bin Li

**Affiliations:** a Shanghai Institute of Applied Physics, Shanghai 201204, People’s Republic of China; b University of Chinese Academy of Sciences, 19 A Yuquan Road, Beijing 100049, People’s Republic of China; c School of Physical Science and Technology, ShanghaiTech University, Shanghai 200031, People’s Republic of China

**Keywords:** X-ray spectrometers, water window, geometric optics, ray tracing, diffraction principle, optical aberration analysis and optimization

## Abstract

The authors report a novel scheme for a grazing-incidence spectrometer forming an excellent meridional flat field in its detector domain to deliver the desired spectral resolution throughout the full designated spectral range, while reducing the sagittal astigmatism substantially to enhance the spectral intensity. The optical properties of the system are thoroughly investigated and optimized, and the detector plane is fitted well to the meridional or sagittal focal curves. The authors demonstrated that a resolving power of 6000–18000 could be achieved within the ‘water window’ (2–5 nm) for an effective meridional source size of 200 µm (r.m.s.), and it would be further improved to 20000–40000 if the source size was confined to 50 µm (r.m.s.).

## Abstract 2

An original scheme has been formulated for a broadband X-ray spectrometer with excellent resolving power throughout the whole spectral range, while the sagittal spot size is well confined to enhance the detection efficiency. The intrinsic optical nature and the most fundamental problems in the system have been comprehensively investigated.

## 1. Introduction   

In recent decades, flat-field spectrometry has been widely used for exploring various intriguing research topics especially in the regions of extreme ultraviolet or soft X-rays, *e.g.* tokamak plasmas (Nakano *et al.*, 1984 ▸), laser-produced warm-dense matter (Schwanda *et al.*, 1993 ▸), stellar-interior properties (Xiong *et al.*, 2011 ▸), magnetic confinement fusion problems (Dong *et al.*, 2011 ▸; Okamoto *et al.*, 2001 ▸), synchrotron radiation light-source development (Koike *et al.*, 2003 ▸; Hague *et al.*, 2005 ▸) and much more. The technique is crucial for providing high spectroscopic resolution in the physical, chemical, photonic and biological sciences.

A flat-field spectrometer employs a grating with varied groove density on a concave substrate to achieve a quasi-flat-field in the detector plane, and then delivers high energy resolution through optimization of the coefficients of the variable line spacing (VLS) for the grating. However, this type of grazing-incidence spectrometer corrects the optical aberrations only in its meridional coordinate and not in the sagittal coordinate, thus it still has significant astigmatism. The meridional rays of the beam are well focused at the detector which is separated from the sagittal focus, displaying a meridionally focused but sagittally diverged two-dimensional spectrograph. Various groups have thus made an effort to achieve better sagittal beam distributions in order to improve the spectral intensity and acquisition efficiency.

Tondello (1979 ▸) demonstrated the stigmatic condition for a spectrometer through the combination of a toroidal mirror and a spherical grating in grazing-incidence geometry. Fan *et al.* (1992 ▸) replaced the toroidal mirror in the design above with a pair of cylindrical and spherical mirrors, and changed the spherical grating with constant groove density to one with VLS. Hettrick *et al.* (1985 ▸) designed an extreme-ultraviolet spectrometer working on a satellite: a pre-focused spherical mirror was utilized to converge the incidence beam beyond a VLS grating to form a virtual source, where nearly normal incidence geometry is applied. This scheme reduced the optical aberration significantly but led to a severe decline in the reflectivity. Nicolosi *et al.* (2005 ▸) developed an optical system simulating a Kirkpatrick–Baez configuration, containing a spherical mirror and a spherical VLS grating to provide the flat-field in the focal plane while restricting astigmatism. Warwick *et al.* (2014 ▸) designed a two-dimensional soft X-ray spectrometer implementing Wolter-type pre-focusing. Vishnyakov *et al.* (2015 ▸) employed a normal-incidence multi-layer spherical mirror to replace the gold-coated mirror in Hettrick *et al.*’s design to enhance the reflectivity and used a better optimized VLS grating to reduce astigmatism; however, the bandwidth of the spectrometer was inevitably limited because of the multi-layer coating. Dvorak *et al.* (2016 ▸) adopted the Hettrick–Underwood spectrometer design using an extra plane mirror to fix the outgoing beam. The defocus and coma of the spectrometer were well compensated, while the mechanical motions of the detector were minimized.

The ‘water window’, spanning the wavelength range of 2–5 nm, is able to provide the excellent contrast imaging for C or O atoms and related structures; this outstanding property could be utilized to image and analyze the biological cells or microstructures *in vitro* and potentially *in vivo*. ‘Water window’ spectroscopy is also a novel probe for material properties and electron energy states. Based on the previous work described above, we designed a novel flat-field spectrometer optimized in the ‘water window’ through systematic investigation of its intrinsic optical nature to exploit its ultimate performance. We have organized this article as follows: §2 introduces numerical simulation and the algorithm to achieve the best meridional focal curve for the spectrometer with various object distances, and then optimizes the sagittal focal curve to well fit the meridional one. In particular, the parameters for evaluating the quality of the meridional or sagittal focal curve are well defined and discussed. §3 presents the systematic design of the proposed spectrometer in detail, using the algorithm in §2 to achieve the desired resolving power in the dispersive coordinate while eliminating astigmatism to improve the spectral intensity. Finally, in §4, we summarize our findings and make some general remarks regarding our design, and discuss the potential research and development in the future.

## 2. Numerical simulation   

**Figure 1:**
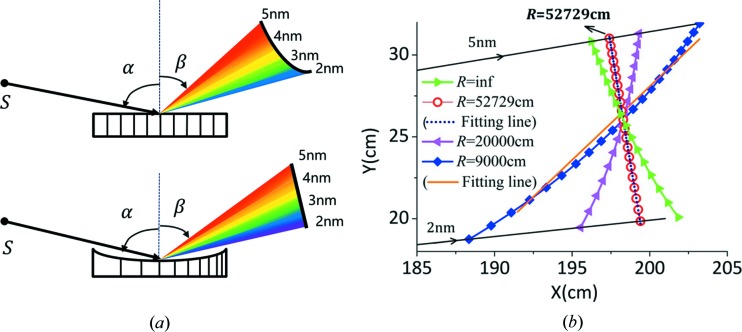
(*a*) A schematic diagram of a VLS grating on a concave substrate to achieve an excellent flat-field condition in the meridional plane within the spectral range 2–5 nm (bottom) is compared with a plane grating with constant groove density whose focal spots for the same spectral range lie in a curved line (top). (*b*) A change of the meridional focal curves of the spectrometer is observed when applying different meridional radii 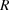, while the identical parameters are as follows: 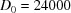
 lines cm^−1^, 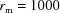
 cm, 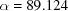
° and the image focal length 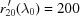
 cm at the center wavelength 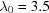
 nm. The straight fitting lines represent the best detector plane for each 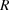, where the value 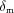 is the magnitude of ‘defocus’ over the whole spectral range [defined in §2.1 and equation (10)]. The fitting lines for 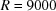 and 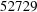
 cm are depicted, while the diffraction beams of 2 nm and 5 nm are shown simultaneously. The calculated value 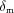 for each case is: 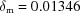
 cm (*R* = infinity), 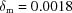
 cm (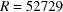
 cm), 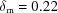
 cm (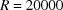
 cm)*,* 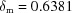
 cm (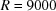
 cm).

As demonstrated in Fig. 1 ▸(*a*), the grating on a concave substrate with VLS groove density is the core of the grazing-incidence spectrometer allowing achievement of an excellent ‘flat field’ in its detector plane, while a plain grating with constant groove density barely achieves that. The coefficients of the VLS grating (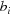) are optimized through the elimination of optical aberrations of various orders in the meridional coordinate, and its groove density can be expressed as (Harada & Kita, 1980 ▸) 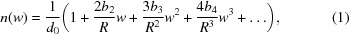
where 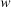 is the meridional coordinate with respect to the center of the grating, 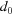 is the groove spacing at 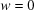 and 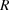 is the meridional radius of the substrate (which is differentiated from the sagittal radius 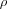 *,* thus the substrate of the grating is actually in a toroidal profile).

Letting 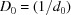, 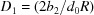, 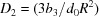, 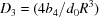, equation (1) is simplified to 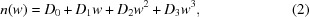
where 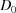 is the grooved line density (the reciprocal of 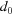) at the center of the grating. According to Fermat’s principle for geometrical optics, the optimal imaging in meridional coordinates could be achieved through zeroing the first-order derivative of the light-path function, thus connecting the light source and the image *via* optics (since the grating is a dispersive optic, various wavelengths are associated with different preferable optical paths) (Samson *et al.*, 1998 ▸). Also, ideally the *F* terms [refer to equation (21)], especially the first few dominants, should satisfy the following equations crossing the wavelength range, 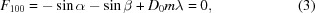


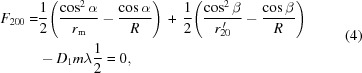


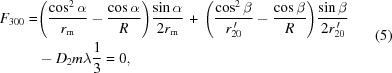


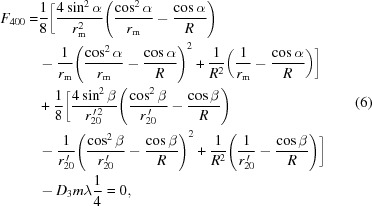
where 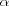 is the incidence angle; 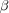 is the diffraction angle; 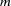 is the order of diffraction (typically 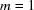 is used in a spectrometer design); 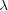 is the wavelength; 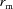 is the meridional object distance; 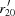 is the meridional image distance; and 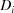 are the VLS coefficients defined in equation (2). More specifically, the equation of 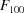 is actually the grating formula; 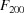 is related to the meridional focus, and could be utilized to characterize the ‘defocus’ over the whole spectral range; and 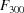 and 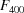 are associated with the ‘coma’ and ‘spherical aberration’, respectively.

### 2.1. Achieving the optimal flat-field in the ‘water window’   

While it would be ideal if equation (4) was satisfied throughout the whole spectral range, this is not possible, so it should be at the center wavelength. Thus, when the meridional object distance 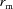, beam incident angle 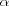, and image distance 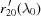 (at the center wavelength 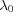) are pre-set, 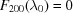 would lead to 
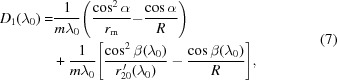
where the first-order VLS coefficient 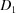 is a function of the meridional radius 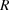. For each value of 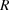, 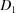 being fixed [according to equation (7)], the meridional image distances for the entire wavelength range can then be calculated, *via* re-arrangement of equation (4), 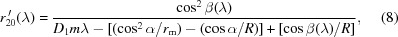
which is wavelength dependent and can be cast into Cartesian coordinates in the principal (*i.e.* meridional) diffraction plane of the VLS grating,
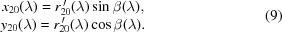
These two-dimensional coordinates [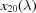, 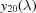] in the principal plane are the theoretical meridional focal spots of various wavelengths, forming an ideal focal curve. The best straight line fitted using these points represents the optimal meridional focal line for the detector (*i.e.* the intersection between the meridional plane and the detector); then the distance from the detector (the corresponding impact spots for various wavelengths) to the grating center 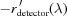 and its orientation in the principal plane can be determined. A root-mean-square value is introduced to characterize how the realistic detector plane approaches the ideal focal plane (or curve) with 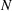 different sampling wavelengths, 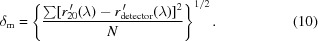
A smaller value of 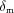 corresponds to a smaller radial separation in between the beam colliding spot on the detector and the actual meridional focal spot, indicating that a better flat-field condition is achieved, *i.e.* the defocusing within the specific wavelength range is minimized.

Implementing a specific set of parameters, for example 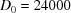
 lines cm^−1^, 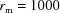
 cm, 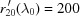
 cm at 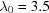
 nm and 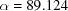
°, the search algorithm for the optimal flat-field within the water window (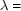 2–5 nm) could be launched. As illustrated in Fig. 1 ▸(*b*), various focal curves, associated with the different values of 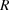 used in the design, are plotted in the same principal plane, which leads to various values of 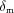, and the minimal value is achieved at the optimal meridional radius 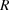 of 52729 cm (red circles). According to the above scheme, each set of parameters would lead to a unique optimal meridional radius of 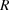 only. This explains the optimization of both the value 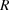 and coefficient 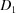, while the object/image distances, beam incident angle and grating groove density (at the center) are fixed. Then, through equations (5) and (6),[Disp-formula fd6] the VLS coefficients of 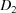 and 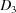 can be derived at the center wavelength 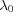.

### 2.2. General discussion for various object distances *r*_m_   

The scheme used to search for the best flat-field condition in the meridional coordinate could be extended to more general cases, *e.g.* implementing different object distances 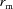, while the values of the grating groove density 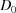, image distance 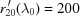
 cm, and the ideal spectral resolving powers of the spectrometer are kept the same as those at 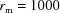
 cm (while the optical aberrations, fabrication or alignment errors in the system were not considered), *via*

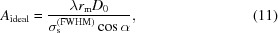
where 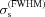 is the size of the light source (at full width at half-maximum) and the other parameters were previously defined. Equation (11) could be used to relate the specific meridional object distance 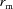 to the corresponding incident angle 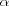. For example, in order to achieve a resolution of 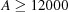 at 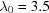
 nm for a source size of 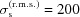
 µm (r.m.s.), we would have 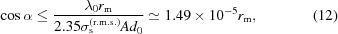
where 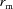 is in units of centimeters. Therefore, the general procedures to achieve the optimal meridional focal curve for a spectrometer are: (i) identify the source size 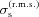, object distance 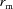, image distance 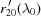 (at 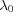), wavelength range and the groove density of the grating 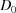; (ii) specify the incident angle 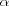 according to equation (12) to achieve the desired spectral resolution at the center wavelength; and (iii) evaluate the defocus 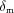 within the whole spectral range using equations (7), (8), (9) and (10) and find out the minimum, thus the optimal meridional radius 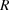 and the associated 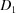 could be determined simultaneously.

**Figure 2:**
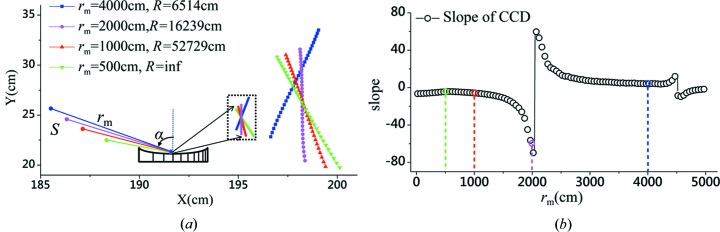
(*a*) The best meridional focal curves (in various colors) for different meridional object distances 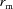 (associated with the various incidence angles to maintain the ideal spectral resolution) are achieved using the scheme discussed in the text, where the image distance 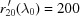
 cm is fixed for various 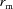. (*b*) The best fitting lines for different 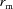 could be identified and regarded as the actual meridional line of the detector plane, its slope in the principal plane of the grating is plot against 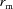 reflecting the orientation of the detector in space, the detector plane is tilted towards the projection of the diffraction beam for smaller 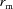 (*e.g.* 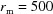
 cm, 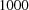
 cm, where the slope of the fitting line is negative) and tilted away from the diffraction beam for larger 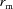 (*e.g.* 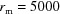
 cm, where the slope is positive). Both (*a*) and (*b*) have the same color code.

Utilizing these procedures, the meridional focal curve, the optimal fitting line and its orientation in the principal plane could be obtained for different meridional object distances (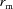). In Fig. 2 ▸(*a*), the best focal curves for various 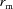 (40, 20, 10 and 5 m) along with an identical image distance 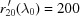
 cm are plotted together at the detector domain. According to equation (12), a smaller 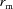 would be correlated to a bigger incidence angle 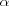 (or a smaller grazing incident angle). Fig. 2 ▸(*a*) clearly illustrates that the optimal meridional focal planes for various 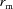 are associated with different inclination angles in the detector domain, and the change in the inclination angle *versus* 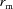 is demonstrated further in Fig. 2 ▸(*b*). When 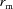 increases, the tilt angle of the detector plane experiences a transition from ‘forward’ (the slope of the fitting line is negative) to ‘backward’ (the slope of the fitting line is positive) at the source distance 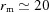
 m, and again from ‘backward’ to ‘forward’ at 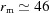
 m [refer to the two thin vertical line segments in Fig. 2 ▸(*b*)].

### 2.3. Optimization of the sagittal focal curve   

In the previous section, we discussed a scheme to achieve the optimal focal curve in the meridional coordinate, now we switch to the sagittal coordinate (for the same wavelength range), 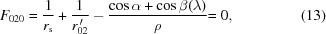
where 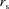 or 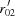 are the sagittal object or image distances, respectively, and 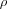 is the sagittal radius of the grating. This leads to 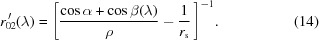
The sagittal focal curve can also be converted into Cartesian coordinates, 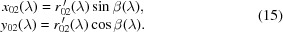
Then the parameter 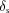, similar to 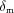, was used to represent the defocus in the sagittal coordinate of the spectrometer,
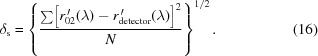
The smaller the value of 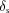, the closer the sagittal focal curve approaches the plane of the detector, and likewise for the meridian focal curve. The search scheme for minimum 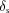 using various series of 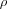 and 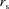 would reduce the astigmatism of the optical system.

**Figure 3:**
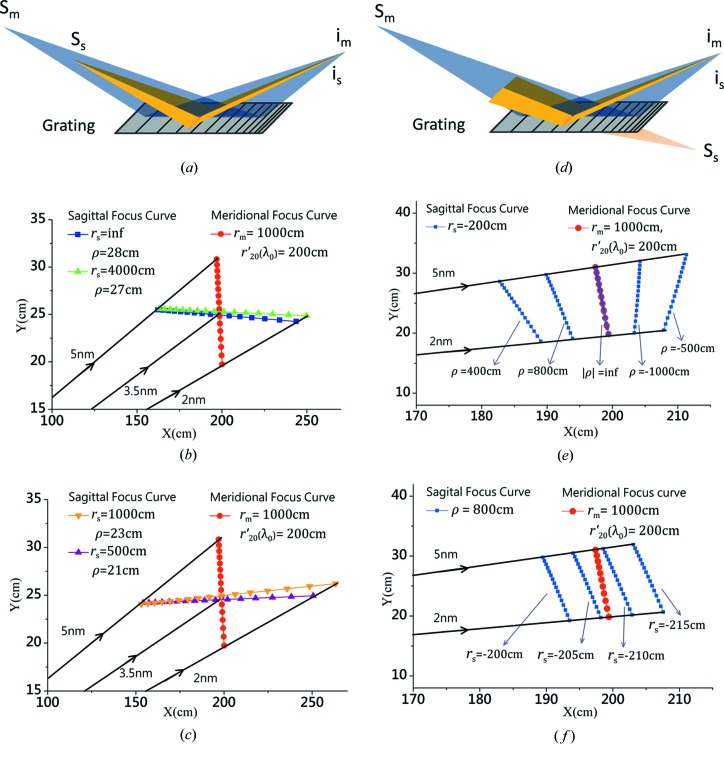
Schematic diagrams to illustrate the light source with different object distances in its meridional (gray) or sagittal (yellow) coordinates, achieving the identical image focal length in the two directions. (*a*) Both the meridional and sagittal object distances are real (*i.e.* 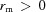 and 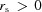). (*d*) The meridional object distance is real, while the sagittal object distance is virtual (*i.e.* 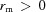 and 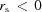). The symbols S_m_ or i_m_ represent the meridional object (or image) point, while S_s_ or i_s_ represent the sagittal object (or image) point. The optimal meridional focal curve (red spots) is presented in each diagram (*b*,* c*, *e* and * f*) as the control group, where 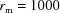
 cm, 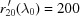
 cm (at the center wavelength), and the other parameters are the same as those for the optimal case in Fig. 1 ▸ (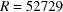
 cm). The sagittal focal curves for various cases are presented in specific plots for comparison. (*b*) The sagittal focal curves for 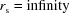, 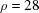
 cm and 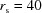
 m, 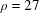
 cm. (*c*) The sagittal focal curve for 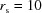
 m, 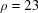
 cm and 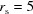
 m, 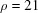
 cm. (*e*) The sagittal focus curves for fixing 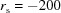
 cm while changing 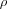. (*f*) The sagittal focus curves for fixing 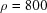
 cm while changing 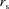.

According to equation (14), the magnitude of 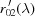 is relevant to both the source distance in sagittal coordinate 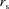 and the sagittal radius of the grating 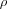. For a more general discussion, we consider the case where the sagittal source point is spatially separated from the meridional one: 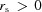 is related to the real sagittal object distance (refer to Figs. 3 ▸ *a*, 3*b* and 3*c*) and 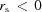 is the virtual case (Figs. 3 ▸ *d*, 3*e* and 3*f*). For each case in Fig. 3 ▸, the meridional focal curve is identical and optimized at 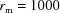
 cm and 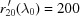
 cm, representing the reference signal (red dots in Figs. 3 ▸ *b*, 3*c*, 3*e* and 3*f*).

For the real sagittal source point (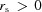) where the sagittal rays of the beam would diverge towards the grating (similar to the meridional rays, Fig. 3 ▸ *a*), the calculated result shows that within the focal region the sagittal beam would intersect with the meridional one at the center wavelength only; while, at the other wavelengths, the spatial separations between the sagittal image focal points and the corresponding meridional image focus are still quite large, indicating that the astigmatism within the spectral range is not significantly reduced, *i.e.* the sagittal image focal curve is still far from being optimized (Figs. 3 ▸ *b* and 3*c*). For the virtual sagittal source point (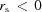), the sagittal beam would converge and achieve the beam waist behind the grating (Fig. 3 ▸ *d*). In Fig. 3 ▸(*e*), where 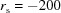
 cm is kept as a constant while the value of 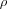 changes, it is observed that both the position and tilt angle of the sagittal focal curve change associatively. When 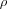 becomes infinite representing a tangentially cylindrical grating, the sagittal focal curve would become a circle with radius 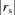 surrounding the center of the grating [refer to equation (14)]. Fig. 3 ▸(*f*) shows the case where 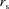 is varied from −200 cm to −215 cm, while 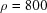
 cm is a constant. The sagittal focal curves are observed to move further away from the grating, but their inclination angles change very little. The above investigation demonstrates that 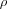 affects both the position and the inclination angle of the sagittal image focal curve, while 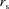 mainly influences the position. Therefore the combination of any arbitrary value of 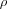 or 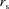 would lead to various shapes and locations of the sagittal focal curve, but the optimal one could always be obtained to well fit with the meridional focal curve.

**Figure 4:**
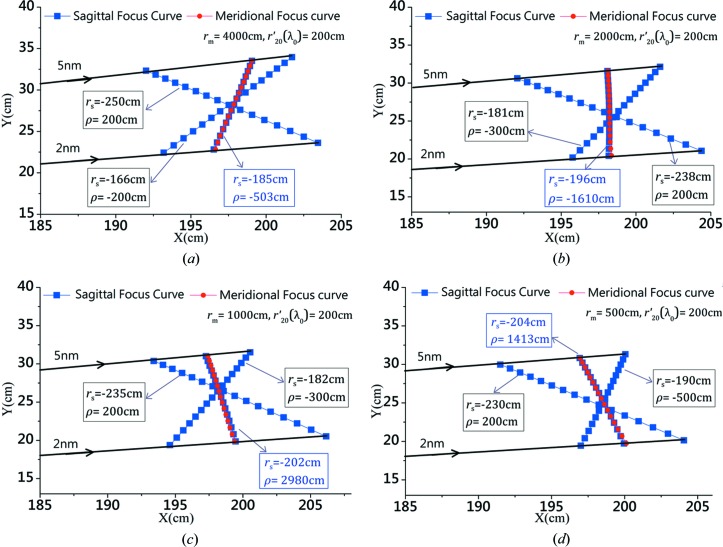
The sagittal focal curves (blue squares) are plotted together with the optimal meridional focal line (red discs) for different configurations (mainly associated with different 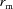, and Table 1 ▸ outlines the explicit list of parameters). For each case, the optimal sagittal focus curve overlaps well with the meridional one, while two non-optimized sagittal focal curves are presented in the same plot for comparison. (*a*) 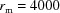
 cm associated with the optimal sagittal parameters: 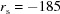
 cm, 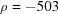
 cm. (*b*) 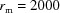
 cm associated with the optimal sagittal parameters: 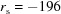
 cm, 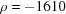
 cm. (*c*) 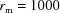
 cm with 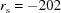
 cm, 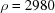
 cm. (*d*) 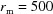
 cm with 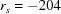
 cm, 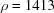
 cm.

Implementing the scheme described in Figs. 3 ▸(*d*), 3(*e*) and 3(*f*), the optimal sagittal focal curves could be identified for various meridional object distances of 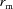 (*e.g.* 4000, 2000, 1000 and 500 cm). In Fig. 4 ▸, the optimal meridional and sagittal focal curves for these four different 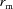 along with identical 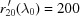
 cm are demonstrated to overlap rather well, where two non-optimized sagittal focal curves are included in each plot for comparison. The key parameters for each case are highlighted, while the explicit list of parameters, including the value of the ‘quality assessment’ (*i.e.* 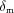 or 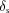), are presented in Table 1 ▸ and discussed in detail in the next section.

## 3. System design   

In the previous section, we formulated a universal scheme for the design of a delicate spectrometer, which could not only achieve decent flat-fields in the meridional coordinate to deliver high spectral resolution (§2.1) for various object distances (§2.2) but also reduced the astigmatism in the sagittal coordinate to enhance the detection efficiency and spectral intensity (§2.3). The flat-field is achieved in the meridional coordinate *via* a VLS grating on a toroidal substrate, while the sagittal object distance 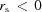 indicates that a virtual light source is preferable in its sagittal direction, which could be realized by using a pre-focusing cylindrical mirror in front of the grating.

**Figure 5:**
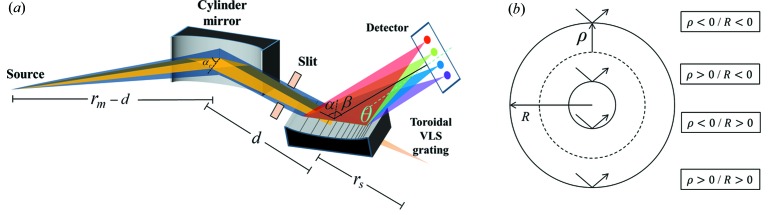
(*a*) Schematic layout of the system design for the sagittally confined flat-field spectrometer in the ‘water window’. (*b*) Options for the toroidal substrate profile of the grating.

Now we propose a realistic spectrometer design in the ‘water window’ possessing the aforementioned merits, which is illustrated in Fig. 5 ▸(*a*). A vertically placed cylindrical mirror is combined with a horizontally placed VLS toroidal grating, with an appropriate spatial separation between them. The incoming beam is focused by the cylindrical mirror horizontally, while propagating down to the grating as a free vertical divergent beam. The source points (with respect to the grating) separate into horizontal or vertical coordinates: the vertical one is located within the meridional (or dispersive) coordinate at the far field, while the horizontal one forms a virtual sagittal source of a converging beam beyond the grating within its non-dispersive coordinate.

In order to achieve this, the radius of the cylindrical mirror should satisfy the following equation,
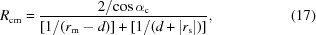
where 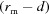 or 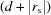 are the effective object or image distances for the cylindrical mirror in its meridional coordinate (where 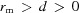 and 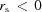 as per previous discussion), 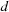 is the distance between the cylindrical mirror and the grating and 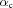 is the incident angle for the cylindrical mirror (which is set equal to the incidence angle of the grating for convenience, *i.e.* 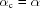).

According to equation (11), the ideal spectral resolution for a spectrometer should be proportional to the object distance, the groove density of the grating and the wavelength, while inversely proportional to the source size. The source size and pixel size of the detector are important parameters for a realistic spectrometer design. In our numerical simulations, we consider a Gaussian beam source profile with a size of 200 µm (r.m.s.) and a divergence angle of 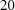
 µrad (r.m.s.), if not otherwise specified, and try to achieve an image size of ∼10 µm at the detector regime to match the realistic pixel size of the detector. In the design of such a spectrometer, we set the spectral resolution above ∼12000 at the center wavelength 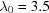
 nm, and optimize the design throughout the ‘water window’ spectrum by using the concrete design parameters listed in Table 1 ▸, for various object distances (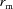) of 40, 20, 10 and 5 m. Two out of four available toroidal surfaces were adopted for the design of the substrate profile of the grating: 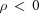, 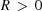 (columns A and B in Table 1 ▸) or 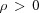, 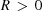 (columns C and D in Table 1 ▸), associated with ‘convex (sagittal)–concave (meridional)’ or ‘concave (sagittal)–concave (meridional)’ surface profiles, respectively (refer to Fig. 5 ▸ *b*), for optimal performance.

In order to calculate the resolving power of the spectrometer, we first evaluate the line width of the diffraction beam distributed at the detector,
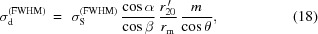
where 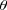 is defined as the angle in between the central diffraction beam and the normal of the X-ray detector (Fig. 5 ▸). It is necessary to point out that a reasonable image-to-object magnification should be implemented to ensure that the line width is greater than the pixel size of the detector to guarantee a realistic resolution, *e.g.* a few µm up to 10 µm.

Then, according to differentiation of the grating formula in equation (3), the spectral line width can be expressed as the image line distribution at the detector,
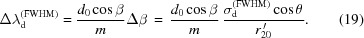
Implementing equations (18) and (19), the spectral line width caused by the light source size can be calculated,
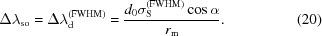
Equations (18), (19) and (20) show how equation (11) is derived (since 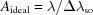), indicating that a Gaussian distribution beam in an aberration-free optical system could achieve the ideal spectral resolution, only limited by the source size. However, in a realistic optical system, the optical aberrations are non-negligible, which would broaden the line width spread of an ideal Gaussian beam distribution substantially. The aberration broadening effect in the meridional coordinate (dispersion direction) can be expressed as 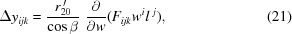
where 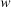 is the illuminated meridional length of the grating, 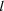 is the illuminated sagittal length, and 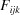 defines the optical aberrations in various orders (the subscripts 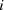 and 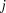 denote the meridional and sagittal coordinates, respectively). Then the spectral distribution broadening caused by the aberration in the system can be evaluated *via* combining equation (19) with equation (21), 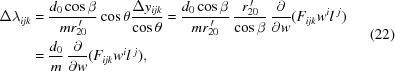
and the dominant meridional aberration terms are (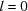)
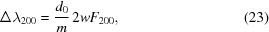


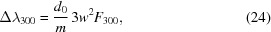


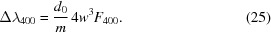
The explicit expressions of 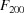, 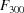 and 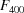 were already given in equations (4), (5) and (6), which are independent of 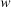 and 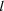. The optical aberrations are set to zero at the center wavelength, *i.e.* 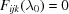, while the defocus 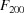 is minimized to achieve an optimal flat field (*i.e.* 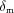 is minimized), the coma 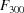 and the spherical aberration 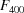 are minimized for the whole ‘water window’ by employing the scheme discussed in §2.1.

Moreover, the optical fabrication error (including the slope error and surface roughness *etc*.) should be taken into account, which broadens the spectral line width by (Thompson *et al.*, 2001 ▸)
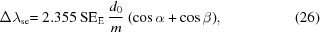
where 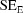 represents the meridional slope error of the grating, while the surface roughness has little impact on the spectral distribution but would strongly influence the beam reflectivity at the surface.

**Figure 6:**
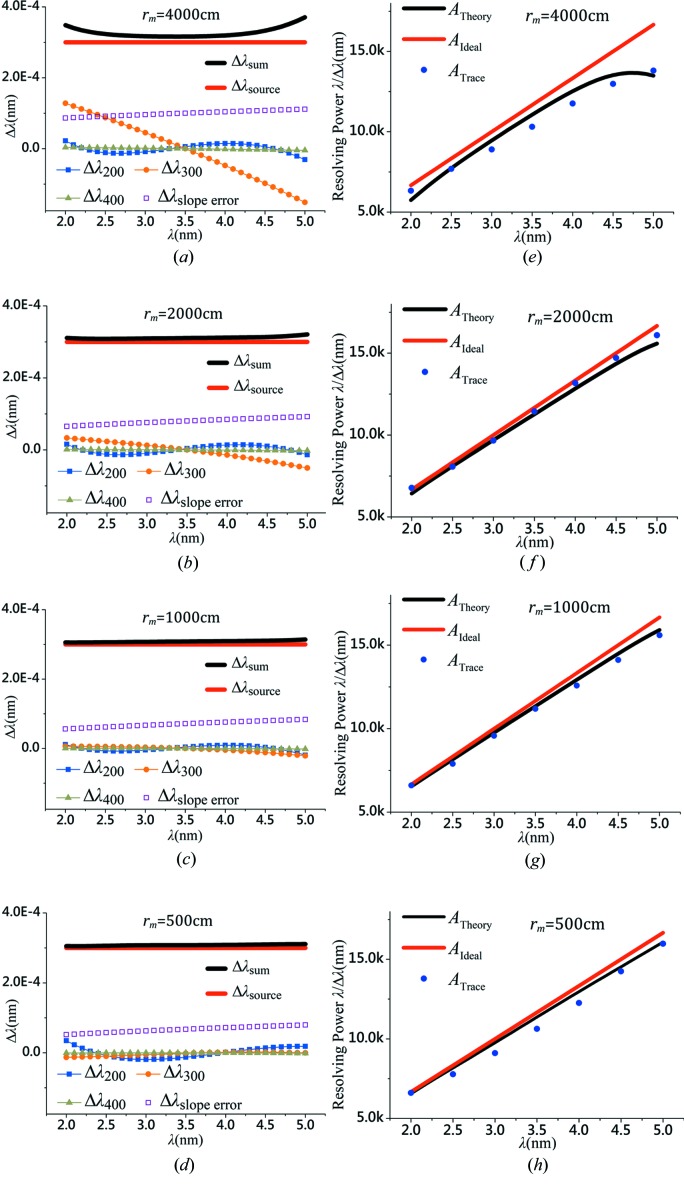
The simulated results of the major factors which influence the resolving power of the spectrometer, including the source size (thick red line), the optical fabrication error (empty squares), the optical aberrations, *i.e.* defocus (filled squares), coma (filled circles), spherical aberration (gray triangles) and overall (thick black lines). The results for various object distances are presented, (*a*) 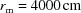, (*b*) 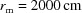, (*c*) 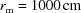 and (*d*) 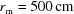; and the image distances for all four cases are identical: 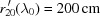. The corresponding resolving powers of (*a*)–(*d*) are calculated and presented in (*e*)–(*h*), respectively, where for each case three types of the spectral resolutions are shown: 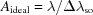, 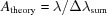 and 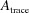 (obtained from the ray-tracing program, see Fig. 7 ▸), for (*e*) 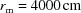, (*f*) 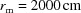, (*g*) 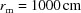 and (*h*) 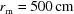.

**Figure 7:**
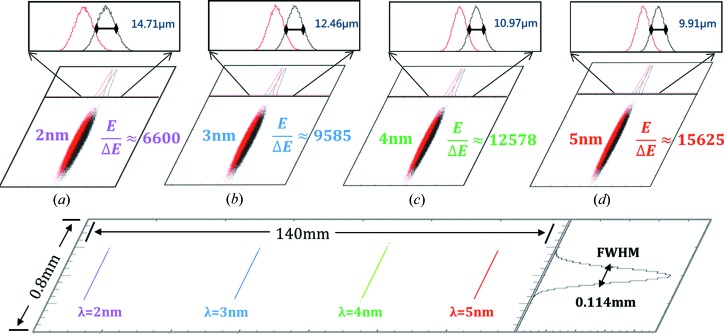
The ray-tracing results for the spectrometer configuration C in Table 1 ▸. The spectral profile distributions at the optimal detector plane for the full wavelength range (2–5 nm) are demonstrated in the lower part of the figure, where the detector dimensions are 140 mm (meridional) × 0.8 mm (sagittal). The ray-tracing results for each wavelength (2, 3, 4 and 5 nm) are presented in (*a*) to (*d*), where an identical ‘domain of interest’ is applied at the detector plane for each of them: the meridional size of the spectrograph is 10–15 µm (FWHM), the sagittal size is about 110 µm (FWHM). Then the resolution power at various wavelengths can be calculated through 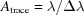: (*a*) 6600 at 2 nm, (*b*) 9500 at 3 nm, (*c*) 12500 at 4 nm and (*d*) 15000 at 5 nm.

The ideal spectral resolution for an aberration-free Gaussian beam was previously given by equation (11). When the overall systematic errors in the spectrometer are inclusive, the resolution can be evaluated (Xue *et al.*, 2015 ▸),
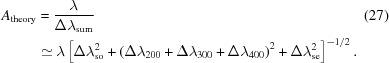



The four defined spectrometer models in Table 1 ▸ (A–D) can be used to calculate the various spectral distribution terms *via* implementing equations (20), (23), (24), (25) and (26), and the results are shown in Figs. 6 ▸(*a*)–6(*d*). The source size term 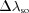 appears to be overwhelming, almost constant within the spectral range (since the source size is assumed to be constant throughout the spectral range). The slope-error term 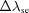 is the second largest component and more or less constant as well. Among the three primary optical aberration terms, the defocus 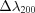 is well confined indicating that an excellent flat-field condition is achieved; the spherical aberration 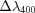 is also quite small, fluctuating around the zero-crossing and negligible; and the value of coma 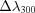 is relatively larger than the other two (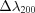 or 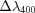) for the configuration A in Table 1 ▸ (Fig. 6 ▸ *a*), and decreases substantially for the configurations B, C and D when the magnification increases (Figs. 6 ▸ *b*–6*d*), *i.e.* 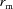 is decreased since 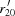 is constant in all cases. The corresponding resolving powers for the configurations in Figs. 6 ▸(*a*)–6(*d*) are calculated and exhibited in Figs. 6 ▸(*e*)–6(*h*), respectively, where in each diagram the ideal spectral resolution 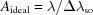, the theoretical resolution 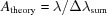, and the result from the ray-tracing program 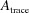 (from Fig. 7 ▸, as discrete spots) are overlaid for comparison.

Additionally, the ray-tracing program for the classical geometric optics (*Shadow*) is utilized to demonstrate the spectral resolution at 2, 3, 4 and 5 nm for configuration C in Table 1 ▸. The bottom part of Fig. 7 ▸ shows the spectral distributions at the optimal detector plane for the whole ‘water window’ (2–5 nm), where the length scales in the meridional (larger) and sagittal (smaller) directions are quite different (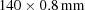). It is worth noting that the sagittal distribution profiles of the four wavelengths are approximately uniform, indicating that the astigmatism of the spectrometer is significantly restricted; for the uncompensated astigmatism case, the sagittal focal size at various wavelengths would be very different. Figs. 7 ▸(*a*)–7(*d*) show the spectral distribution and resolution at each individual wavelength (2, 3, 4 and 5 nm in terms of 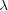 and 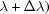, which are traced in a 400 µm × 400 µm square detector domain. The FWHM widths for each wavelength in meridional coordinates, illustrated in specific sub-plots, can be used to evaluate the realistic spectral resolution 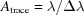, and the results are presented in Fig. 6 ▸.

## 4. Discussion   

In summary, we report the use of a novel spectrometer design in combination with a sagittal pre-focusing cylindrical mirror and a toroidal VLS grating. This design could not only provide a decent flat field in the meridional coordinate to achieve the desired resolving power for the whole spectral range but also greatly reduced astigmatism in the sagittal coordinate to enhance the spectral intensity. Our main findings in the current research are: (i) with various meridional object distances 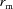 employed in the spectrometer design, the specific incident angle 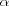 correlated to each of the 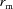 could be determined to deliver a constant spectral resolution; (ii) for each 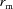, there is only one unique set of meridional radii *R* along with the ‘defocus’ correction coefficient 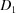 (the lowest order of the VLS coefficients) for the grating, which could achieve the optimal meridional focal curve 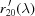 throughout the spectral range; meanwhile, the ‘coma’ and ‘spherical aberration’ of the system could be significantly reduced by optimizing the VLS coefficients 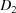 and 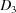; (iii) then the best sagittal focal curve (which overlaps the meridional one quite well) could be achieved through optimizing the radius of a sagittal pre-focusing cylindrical mirror 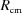 and the sagittal radius of the grating 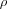.

Thus, the optical aberrations present in a grazing-incidence X-ray spectrometer could be well compensated by the scheme described above. The idea of separating the object (or source) points into its meridional or sagittal coordinates provides a high degree of freedom for selecting and optimizing the parameters in the design *via* a rather simple simulation algorithm. Here, we implemented the scheme for a spectrometer in the ‘water window’ with a large source distance (*i.e.* the image-to-object magnification is less than 1), which is applicable to a light source split from the beamline of synchrotron radiation or a free-electron laser. The scheme is not limited to this and could also be employed in the design of a compact spectrometer. Furthermore, we have the flexibility to pursue an even higher resolving power, by inserting a meridional confinement slit into the incident beamline to achieve a smaller effective source size for the spectrometer (refer to Fig. 5 ▸ *a*). However, the smaller source size would correspond to a smaller imaging line width at the detector, which needs to be greater than the detector’s pixel size to guarantee the spectral resolution. More details regarding this can be found in the supporting information.

Although we mainly discuss the spectrometer design in the ‘water window’, the algorithm has universal adaptability, which could easily be extended to a much broader photon-energy (or wavelength) range through an appropriate modification to the design parameters. It is also feasible to utilize the scheme to develop a high-performance grating monochromator simply by putting a fine slit across the focal curve of the diffraction beam.

## Supplementary Material

Supporting figures S1 and S2. DOI: 10.1107/S160057751800468X/yi5050sup1.pdf

## Figures and Tables

**Table 1:** The design parameters of the optimized spectrometer for four different source distances The schematic layout is presented in Fig. 5 ▸.

Configuration	A	B	C	D
Cylindrical mirror				
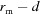 (cm)	3960	1960	960	460
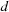 (cm)	40	40	40	40
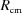 (cm)	6964	13779	25287	41718
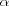 ( 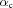 ) (°)	86.494	88.248	89.124	89.560
				
Toroidal VLS grating				
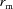 (cm)	4000	2000	1000	500
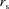 (cm)	−185	−196	−202	−204
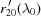 (cm)	200	200	200	200
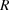 (cm)	6514	16239	52729	Infinity
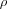 (cm)	−503	−1594	2980	1413
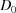 (lines cm^−1^)	24000	24000	24000	24000
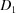 (lines cm^−2^)	205.1	224.8	235.1	240.15
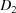 (lines cm^−3^)	1.693	1.698	1.749	1.748
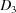 (lines cm^−4^)	0.017	0.011	0.011	0.012
				
Footprint (FWHM) on the grating surface				
*w* (cm)	3.158	3.499	4.350	6.711
*l* (cm)	0.1619	0.0858	0.0514	0.0383
				
Slope errors				
SE_m_ (µrad)	0.5	0.5	0.5	0.5
SE_s_ (µrad)	2	2	2	2
				
Quality assessment				
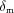 (cm)	0.0033	0.0031	0.0018	0.0029
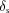 (cm)	0.0302	0.0416	0.0749	0.0484
