# Supplementary material for: A sagittally confined high-resolution spectrometer in the ‘water window’
Source: J Synchrotron Radiat. 2018 Apr 25;25(Pt 3):738–47. doi: 10.1107/S160057751800468X (PMC5929356; doi:10.1107/S160057751800468X)
Supplement: Supplementary file 1 [file s-25-00738-sup1.pdf]

JOURNAL OF SYNCHROTRON RADIATION

Volume 25 (2018)

Supporting information for article:

A sagittally-confined high-resolution spectrometer in "water window"

Zhuo Li and Bin Li

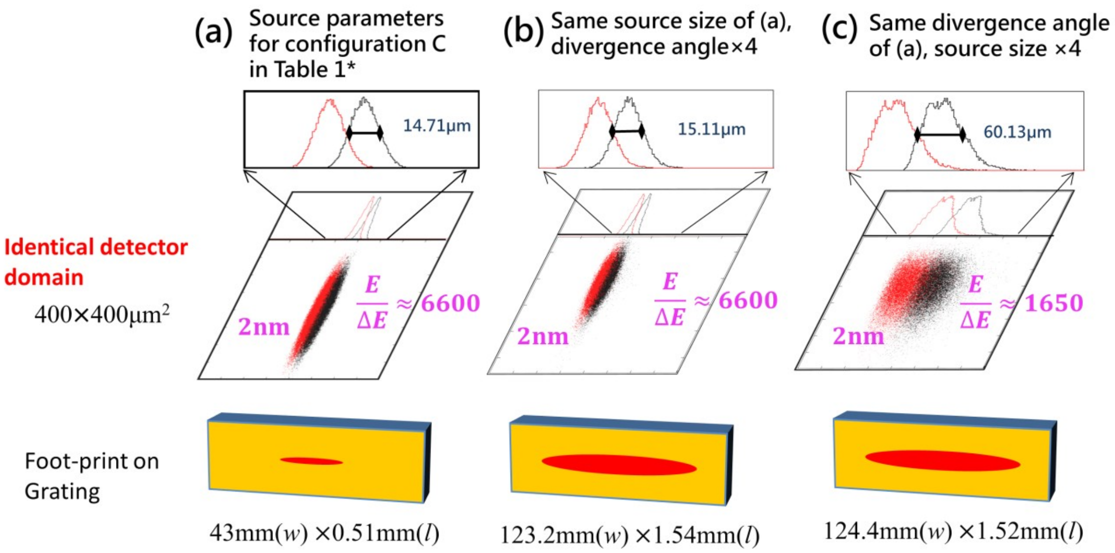

Figure S1 Additional ray-tracing results for the spectrometer configuration (C) in Table.1 of the main text: (a) by using the identical parameters, light source size of 200 μm (r.m.s), and divergence angle of 20 μrad (r.m.s), (b) the same source size while the divergence angle increases four times, (c) the same divergence angle while the source size increases four times. For each case, the corresponding beam foot-print on the surface of the grating is illustrated underneath, where w or l represents the Meridional or Sagittal coordinate respectively, and the values are given as the FWHM size of the beam.

According to the previous discussion, the light source size of 200 μm (r.m.s), and divergence angle of 20 μrad (r.m.s) are adopted for the spectrometer design. While the parameters of the configuration (C) in Table 1 (of the main text) are implemented, the spectral resolution of ~6600 could be achieved at $\lambda = 2$ nm, and the beam footprint on the grating is 43.5 mm (w) × 0.51 mm (l) (figure.S1(a)). If the beam size is kept the same while the divergence angle increases four times, i.e. to 80 μrad (r.m.s), the ray-tracing indicates that the footprint on the grating increases three times approximately while the spectral resolution remains as its original value (figure.S1(b)). However, if the beam divergence is kept the same while the source size increases four times, i.e. to 800 μm

(r.m.s), the ray-tracing exhibits that the resolving power decreases substantially down to ~1650 (figure.S1(c)). The comparison here demonstrates that the optical aberrations in the spectrometer are well compensated and corrected, so increasing the divergence angle wouldn't influence the resolving power, while only debase the quality of the spectrograph a bit. The size of the light source is the dominant factor restricting the resolution power, which could be further improved via narrowing down the source size, especially the meridional one.

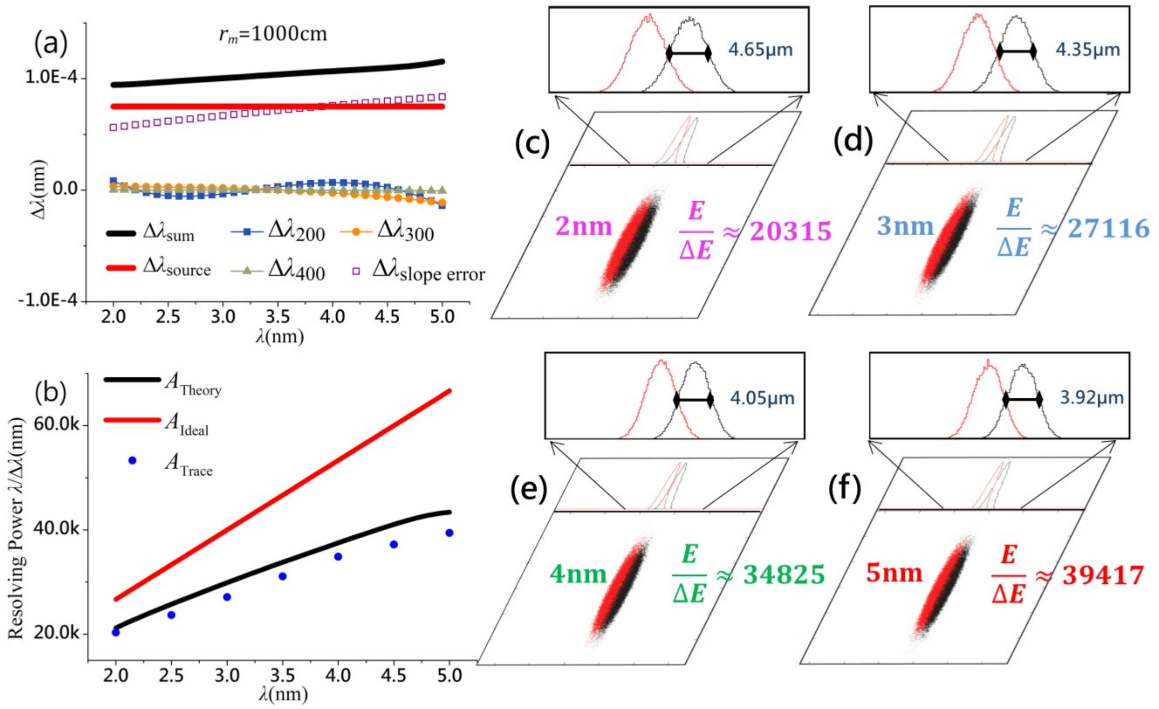

Figure S2 The ray-tracing results for the spectrometer configuration (C) in Table.1, but with a much smaller meridional source size of 50 μm (compared to figure. 7).

Furthermore, as illustrated in figure. 5(a) of the main text, if a confinement slit is inserted into the incident beam path to narrow down the meridional beam size, which simulates to decreasing its effective source size, the spectral resolution would apparently be enhanced further. While the source size (200 μm) is changed to 50 μm , 'Footprint' at the grating surface, Quality Assessment values, and the spectral resolving powers in Table.1(C) could be recalculated by the scheme described in Section 3. As demonstrated in figure. S2, the theoretical spectral resolution (A_{Theory} or A_{Trace}) could be improved substantially to 20000-40000 within the "water window" spectrum (2-

5nm), however, it deviates more from the ideal resolution (A_{ideal}) compared to the previous case (figure. 6). This implies that the optical fabrication errors cause relatively stronger influence on a smaller source size in the spectrometer design. Nevertheless, this also demonstrates that, upon scarifying certain amount of beam flux, the spectrometer has potential to further increase the resolution power throughout the spectral range.
